# Trichomonas vaginalis, HIV, and African-Americans.

**DOI:** 10.3201/eid0706.010603

**Published:** 2001

**Authors:** F Sorvillo, L Smith, P Kerndt, L Ash

**Affiliations:** Department of Epidemiology, School of Public Health, University of California, Los Angeles, 90024, USA. fsorviill@ucla.edu

## Abstract

Trichomonas vaginalis may be emerging as one of the most important cofactors in amplifying HIV transmission, particularly in African-American communities of the United States. In a person co-infected with HIV, the pathology induced by T. vaginalis infection can increase HIV shedding. Trichomonas infection may also act to expand the portal of entry for HIV in an HIV-negative person. Studies from Africa have suggested that T. vaginalis infection may increase the rate of HIV transmission by approximately twofold. Available data indicate that T. vaginalis is highly prevalent among African-Americans in major urban centers of the United States and is often the most common sexually transmitted infection in black women. Even if T. vaginalis increases the risk of HIV transmission by a small amount, this could translate into an important amplifying effect since Trichomonas is so common. Substantial HIV transmission may be attributable to T. vaginalis in African-American communities of the United States.


### *Trichomonas vaginalis*, HIV, and African-Americans

**Frank Sorvillo,* Lisa Smith,* Peter Kerndt,† Lawrence Ash***
*University of California at Los Angeles, Los Angeles, California; and
†Department of Health Services, Los Angeles County, Los Angeles, California

*Trichomonas vaginalis* may be emerging as one of the most important cofactors in amplifying HIV transmission, particularly in African-American communities of the United States. In a person co-infected with HIV, the pathology induced by *T. vaginalis* infection can increase HIV shedding. *Trichomonas* infection may also act to expand the portal of entry for HIV in an HIV-negative person. Studies from Africa have suggested that *T. vaginalis* infection may increase the rate of HIV transmission by approximately twofold. Available data indicate that *T. vaginalis* is highly prevalent among African-Americans in major urban centers of the United States and is often the most common sexually transmitted infection in black women. Even if *T. vaginalis* increases the risk of HIV transmission by a small amount, this could translate into an important amplifying effect since *Trichomonas* is so common. Substantial HIV transmission may be attributable to *T. vaginalis* in African-American communities of the United States.

*Trichomonas vaginalis* is a protozoan parasite transmitted principally through vaginal intercourse. Infection with the organism, while frequently asymptomatic, can cause vaginitis in women and urethritis in men. Despite a relative paucity of studies on the prevalence and incidence of trichomoniasis, recent publications suggest that *T. vaginalis* is one of the most common sexually transmitted infections (STIs) in the United States, with an estimated 5 million new cases occurring annually (1). Although the organism appears to be highly prevalent and has a widespread geographic distribution, *Trichomonas* has not been the focus of intensive study nor of active control programs. This neglect is likely a function of the relatively mild nature of the disease (2), the lack of effect on fertility, and the historic absence of association with adverse birth outcomes (although recent data suggest a possible causal role in low birth weight and prematurity [3]). However, *Trichomonas* may play a critical and underrecognized role in amplifying HIV transmission (4). We present the rationale to support the hypothesis that *T. vaginalis* may be an important cofactor in promoting the spread of HIV and, in some circumstances, may have a major impact on the epidemic dynamics of HIV in African-American communities.

### Biologic Rationale

### Expanding the Portals of Entry and Exit

*T. vaginalis* infection typically elicits an aggressive local cellular immune response with inflammation of the vaginal epithelium and exocervix in women and the urethra of men (5). This inflammatory response induces a large infiltration of leukocytes, including HIV target cells such as CD4+ bearing lymphocytes and macrophages to which HIV can bind and gain access (6,7). In addition, *T. vaginalis* can frequently cause punctate mucosal hemorrhages (8). In an HIV-negative person, both the leukocyte infiltration and genital lesions induced by *Trichomonas* may enlarge the portal of entry for HIV by increasing the number of target cells for the virus and allowing direct viral access to the bloodstream through open lesions. Similarly, in an HIV-infected person the hemorrhages and inflammation can increase the level of virus-laden body fluids, the numbers of HIV-infected lymphocytes and macrophages present in the genital contact area, or both. The resulting increase of both free virus and virus-infected leukocytes can expand the portal of exit, thereby heightening the probability of HIV exposure and transmission to an uninfected partner. Increased cervical shedding of HIV has been shown to be associated with cervical inflammation (9), and substantially increased urethral viral loads have been documented in men with *Trichomonas* infection (10). In addition, *T. vaginalis* has the capacity to degrade secretory leukocyte protease inhibitor, a product known to block HIV cell attachment; this phenomenon may also promote HIV transmission (11). Moreover, since most patients with *Trichomonas* infection are asymptomatic or mildly symptomatic (12), they are likely to continue to remain sexually active in spite of infection. Studies suggest that approximately 50%-70% of persons with *T. vaginalis* have subclinical infection (12).

### Empiric Evidence Implicating Trichomonas in HIV Transmission

Data from studies conducted in Africa have shown an association between *Trichomonas* and HIV infection, suggesting a two- to threefold increase in HIV transmission

Address for correspondence: Frank Sorvillo, Department of Epidemiology, School of Public Health, UCLA, Box 951772, Los Angeles, CA 90024, USA; fax: 714-816-9099; e-mail: fsorvill@ucla.edu

(4,13,14). A cross-sectional study conducted among 1,209 female sex workers in the Ivory Coast found an association between HIV and *Trichomonas* infection in bivariate analysis (crude odds ratio 1.8, 95% confidence intervals 1.3, 2.7). In another cross-sectional study performed in Tanzania among 359 women admitted to a hospital for gynecologic conditions, *Trichomonas* was more common in women with HIV infection in multivariate analysis (odds ratio 2.96, no confidence intervals provided, p<0.001). While such cross-sectional studies are limited by the issue of temporal ambiguity, i.e., lack of information on whether *Trichomonas* infection preceded HIV, these preliminary findings were subsequently reinforced in a single prospective study from Zaire (4). This study, in which 431 HIV-negative female prostitutes were evaluated over time, found that prior *Trichomonas* infection was associated with a twofold increased rate of HIV seroconversion in muiltivariate analysis.

### Data on the Prevalence of *T. vaginalis* among U.S. Women

Information on the occurrence of *T. vaginalis* infection in the United States is meager. Trichomoniasis is not a reportable condition in most health jurisdictions, and prevalence surveys for STIs often do not include attempts to recover *Trichomonas*. In addition, the relatively few published studies with information on the prevalence of *T. vaginalis* infection have generally been conducted among highly selected populations, typically included only women, or were limited by small numbers of participants. Frequently these studies were not conducted with the primary purpose of assessing the prevalence of *Trichomonas*. Moreover, many of these studies have often used diagnostic techniques with relatively low sensitivity such as wet mount, stained preparations, or Papanicolaou (PAP) smear. Wet mount, the most commonly used method, has an estimated sensitivity of 58% when compared with culture (15); the sensitivity of PAP smear is approximately 57%. The accuracy of these techniques is dependent on the experience of the microscopist, and sensitivities may vary widely (15). The sensitivity of culture when compared with polymerase chain reaction (PCR) has been estimated to be 70% (16). Such highly sensitive PCR and related techniques are not routinely used nor readily available for *Trichomonas* as for other STIs (17). As a result of suboptimal laboratory methods, studies of *T. vaginalis* have often substantially underestimated the prevalence of infection. In spite of this, levels of infection have typically been high, with reported overall prevalences ranging from 3% to 58% and an unweighted average across studies of 21% (18-37).

Table 1 lists published reports on the occurrence of *T. vaginalis* infection among women conducted among U.S. populations from 1964 through 1999. Although not

Table 1. Studies of the prevalence of *Trichomonas vaginalis* infection in women, United States, 1964–1997

| Year[a] | Location (ref) | N | Population | Trichomonas prevalence (%) | Diagnostic method(s) |
|---|---|---|---|---|---|
| 1996-97 | New York (18) | 213 | Incarcerated | 47 | culture |
| 1995-97 | St. Louis (19) | 143 | HIV clinic | 11 | wet mount |
| 1993-95 | 4 cities (20) | 1,285 | HIV infected and high risk | 11 | wet mount |
| 1994 | New York (23) | 1,404 | Inner city | 20 | not provided |
| 1992 | Baltimore (24) | 279 | STD clinic | 26 | culture |
| 1990-94 | New York (37) | 677 | HIV and community clinics | 22 | culture |
| 1901-93 | Southeastern city (21) | 650 | Adolescent health clinics | 3 | culture |
| 1986 | 5 cities (27) | 13,816 | Antepartum women | 13 | culture |
| 1990-91 | New York (22) | 372 | Inner city | 27 | culture |
| 1989-90 | New York (25) | 1,401 | OB/GYN clinics | 20 | culture |
| 1989 | Baltimore (26) | 3,005 | Cancer screening | 25 | wet mount |
| 1987-88 | Denver (36) | 5,681[b] | STD clinic | 11 | wet mount |
| 1984-86 | Birmingham (28) | 818 | STD clinic | 21 | wet mount |
| 1985 | San Francisco (29) | 171 | Adolescent clinic | 11 | wet mount/PAP[c] |
| 1982 | Baltimore (30) | 115 | Pregnant adolescents | 34 | culture |
| 1981 | Seattle (31) | 80 | Juvenile detention | 48 | wet mount |
| 1980 | Providence (32) | 500 | Student health center | 3 | culture |
| 1979-80 | Storrs (33) | 383 | GYN clinic | 19 | wet mount/PAP |
| 1971 | Oregon (34) | 338 | State school/adolescents | 35 | Gram stain |
| 1964 | Philadelphia (35) | 27,392 | Cancer screening | 16 | PAP |

[a]Year of study (or publication).
[b] Number of visits.
[c]Papanicolaou smear; STD = sexually transmitted disease; OB/GYN = obstetrics/gynecology.

necessarily complete, a comprehensive search through MEDLINE and review of articles yielded only 20 reports during this 35-year period. Evaluated populations have included such groups as sexually transmitted disease (STD) clinic patients, inner-city populations, pregnant women, university students, adolescents, incarcerated populations, and women with HIV infection.

### Data on the Incidence of *T. vaginalis* in the United States

Even fewer studies have assessed the incidence of trichomoniasis in the United States. In a study conducted from 1992 to 1995 among a cohort of 212 women with HIV in Los Angeles County, *Trichomonas* infection was the most frequently identified sexually transmitted disease and was found in 37 (17.4%) women, representing a crude incidence rate of 14.1 per 100 person-years' experience (38). The crude rate was highest in black women (69.0 per 100 person-years). A recent prospective study conducted from 1990 to 1998 in New Orleans, which followed women co-infected with HIV and *T.* vaginalis, documented high rates (16.1 per 100 person-years) of *Trichomonas* re-infection (39). Among a predominantly black group of HIV-infected and high-risk women followed in New York City from 1990 to 1994, *T. vaginalis* was the most frequent incident STI (37).

### Prevalence of *T. vaginalis* among Men in the United States

Very few published studies have assessed the prevalence of *T. vaginalis* among men and, as is the case for women, these studies typically have included relatively small samples from selected populations. Often data on race-specific prevalences are not provided. Among men attending an STD clinic in Seattle-King County from 1987 to 1990, 6% of 300 randomly selected men were infected with *Trichomonas* by culture technique; 22% of 147 contacts to women with *T. vaginalis* were also positive (40). In a study published in 1995 conducted in Richmond, California, 12% of 204 male patients from an STD clinic were culture positive for *T. vaginalis* (41). Among 454 consecutive men attending an STD clinic in Denver in 1998, 2.8% were found to be infected by a culture method (42). In a small-scale study published in 1991 among 16- to 22-year-old black men enrolled in an inner-city residential youth job-training program, *Trichomonas* was recovered from 55% of 85 participants and was the most common STI identified (43). Data on race-specific prevalences of *Trichomonas* infection among U.S. males are not available. We are unaware of any published reports that have assessed the prevalence of *T. vaginalis* in males and females. While the separate studies we have cited suggest that *Trichomonas* may be more common in women in the United States, the data are so limited and potentially biased that any such conclusions must be made cautiously.

### Race and *Trichomonas*

Table 2 presents data, where available, on the prevalence of *Trichomonas* among women, by race, in the United States. In each study that has presented information on race/ethnicity, the prevalence of *Trichomonas* has been highest in African-Americans (23%-51%), ranging from approximately 1.5 to nearly 4 times greater than other racial/ethnic groups. In several studies in which very high prevalences of infection were observed, the population consisted exclusively or predominantly of African-Americans. This racial finding, consistent across studies, is unlikely to be artifactual.

Table 2. Prevalence of *Trichomonas vaginalis* among women, by race, United States

| City (ref) | Overall *Trichomonas* prevalence (%) | *Trichomonas* prevalence in blacks | *Trichomonas* prevalence in non-blacks | OR[a] |
|---|---|---|---|---|
| New York (18) | 47 | 51 | 35 | 1.6 |
| San Francisco (29) | 11 | 28 | 9 | 3.7 |
| 5 cities (27) | 13 | 23 | 6 | 4.4 |
| Philadelphia (35) | 16 | 30 | 11 | 3.6 |
| New York (22) | 27 | population 92% black | | |
| New York (25) | 20 | population 83% black | | |
| Baltimore (24) | 26 | population 96% black | | |
| New York (23) | 20 | population 90% black | | |
| Baltimore (26) | 25 | population 100% black | | |
| Birmingham (28) | 21 | population 89% black | | |
| Providence (32) | 3 | population 87% black | | |

[a] Estimated odds ratio.

Several factors may explain the apparent elevated rate of trichomoniasis in black women. This phenomenon may indicate a high prevalence of *Trichomonas* infection among the sex partners of these women. Although a study in Washington, D.C., observed a high prevalence of *T. vaginalis* (55%) among young, inner-city, black men (43), data on race-specific rates of *Trichomonas* infection in men are lacking. The association with black race may also reflect decreased use of barrier protection in this population. Studies indicate that African-American males are less likely to use condoms than men of other racial groups because of a higher frequency of condom breakage and slippage (44) and a reported decrease in sexual fulfillment (45). Alternatively, it is possible that practices such as douching, which is reportedly more common in black women (46) and can increase susceptibility to other STIs (47), could predispose to trichomoniasis and explain the observed racial association. Increased prevalences of *Trichomonas* infection could also reflect lack of access to care and distrust of the health-care system, which could manifest as failure to seek care, noncompliance with treatment recommendations, and hesitation to refer partners for treatment. Drug use and its association with high-risk sexual behaviors, including trading sex for money or drugs, may also explain the racial differences in the occurrence of *Trichomonas*. In addition, compared with other racial and ethnic groups, a greater proportion of blacks are unmarried, divorced, or separated (48), and unmarried status is itself a risk marker for STIs (49). It is also conceivable that a genetic or racial-based heightened susceptibility to *T.vaginalis* exists in African-Americans; however, such a phenomenon has not been recognized. Finally, the observed racial disparity could reflect strain differences of *Trichomonas*. For example, if the strains that infect African-Americans are more likely to produce chronic, persistent infection of longer duration, higher prevalences would be observed. However, this hypothesis has not been studied.

### *Trichomonas* Compared with Other STIs in African-American Women

Table 3 lists studies comparing the prevalence of *T. vaginalis* infection with that of other STIs among black women in the United States. In each study *Trichomonas* was the most commonly identified STI, exceeding both *Chlamydia trachomatis* and *Neisseria gonorrhoeae* in prevalence. While the optimal tests for detecting *C. trachomatis* and *N. gonorrhoeae* were not always used in these studies, neither were highly sensitive tests used for the diagnosis of *Trichomonas*.

### Discussion and Implications

The HIV/AIDS epidemic is a heterogeneous one, impacting communities and subpopulations in disproportionate ways. In many jurisdictions in the United States, HIV is increasingly affecting low-income groups, particularly African-Americans and women. We suggest that part of this phenomenon may result from the amplifying effect of *T.vaginalis*. Several aspects of the biology and epidemiology of *Trichomonas* suggest that this long-neglected protozoan may play an important role in HIV transmission dynamics. A compelling biologic rationale suggests that the pathology caused by *Trichomonas* enhances the efficiency of HIV transmission. In addition, *T. vaginalis* infection is often asymptomatic, and affected persons are likely to continue to engage in sexual activity. This strong biologic plausibility is supported by empiric studies from Africa documenting that *Trichomonas* may increase HIV transmission by two- to threefold. Moreover, although imperfect, the available data suggest that *T. vaginalis* is a highly prevalent infection, particularly among African-American women in urban communities of the United States. Given the evidence that *T.vaginalis* likely promotes HIV infection, the apparent high level of *Trichomonas* infection in black women is cause for concern. Even if *T. vaginalis* increases the risk of HIV transmission by a small or modest amount, it may translate into a sizable population effect since *Trichomonas* is so common. To illustrate this, we present population-attributable risk curves, or the level of HIV transmission that would be attributable to *T. vaginalis*, at varying prevalences of *Trichomonas*, given the assumption of an increased relative risk of HIV infection of 2 or 3 (Figure). As the figure illustrates, if *Trichomonas* amplifies HIV transmission by twofold and the prevalence of *T. vaginalis* in a community is 25%, one fifth (20%) of HIV transmission in that population would be attributable to *Trichomonas*. This has important implications for HIV prevention. Reduction in the prevalence of *Trichomonas* could translate into substantial decreases in HIV transmission. Effective, inexpensive single-dose therapy (2 g oral metronidazole) is available for the treatment of *T. vaginalis* infection. It may not be hyperbole to suggest that *Trichomonas* infection may be more readily modifiable than sexual behavior in some high-risk groups. Trials in Tanzania have demonstrated the benefit of reduced HIV incidence in communities receiving aggressive STD control intervention (50).

While convincing data suggest that other STDs, including both ulcerative and inflammatory infections, promote HIV transmission (51), available evidence suggests that *T.vaginalis* is the most common STI in African-American women and therefore may play a more prominent role than other STIs in augmenting the spread of HIV in this high-risk group.

Additional studies to evaluate the prevalence and incidence of *T. vaginalis* and to determine risk factors for infection in both men and women are needed. Moreover, given the paucity of data and the potential importance of *Trichomonas*, consideration should be given to requiring mandatory reporting of *T. vaginalis* infection. Efforts to further evaluate

Table 3. Studies comparing the prevalence of *Trichomonas vaginalis* infection with that of other sexually transmitted infections among black women in the United States

| Year | City (ref) | Trichomonas (%) | Chlamydia (%) | Gonorrhea (%) |
|---|---|---|---|---|
| 1996 | New York (18) | 51 | 9 | 5 |
| 1994 | New York (22) | 27 | 7 | 2 |
| 1994 | New York (23) | 20 | 15 | no data |
| 1992 | Baltimore (24) | 26 | 21 | 14 |
| 1990 -94 | New York (37) | 22 | 6 | 1 |
| 1985 | San Francisco (29) | 28 | 25 | no data |

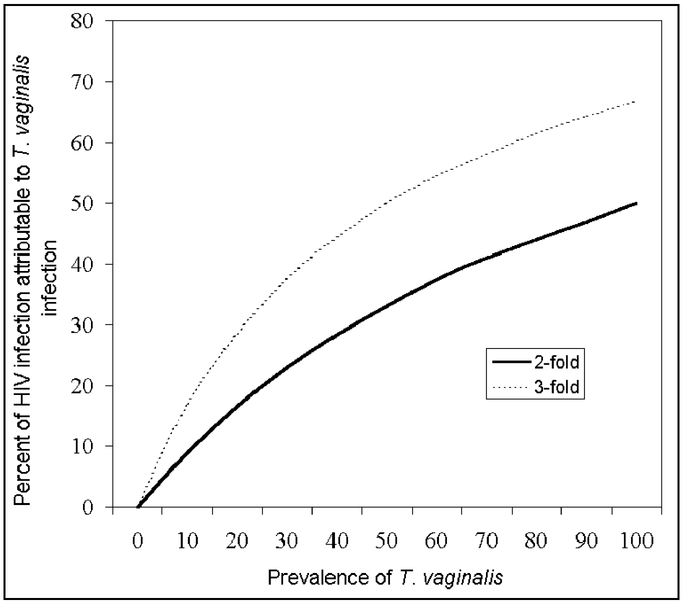

Figure. Hypothetical level of HIV transmission attributable to *Trichomonas vaginalis* at varying prevalences of *Trichomonas* infection and assuming that *T. vaginalis* infection amplifies HIV infection by two- or three-fold.

the interactions between *T. vaginalis* and HIV, particularly in an industrialized country setting, would also seem warranted. However, given the lower rates of heterosexual transmission, such studies would be expensive and require a large sample. Nevertheless, we believe that current information is compelling enough to warrant considering implementation of efforts to identify and treat persons with *T. vaginalis* infection, particularly African-Americans, in areas of overlapping HIV and *T. vaginalis* epidemics. Screening programs using self-collected vaginal swabs (52) for culture may be a reasonable method for such an effort. An alternative approach would be to first use wet mount examination, which is relatively easy and inexpensive but lacks sensitivity, followed by culture for specimens that are negative on wet mount. Recent development of sensitive and specific urine-based diagnostic techniques can enhance both the yield and ease of screening efforts (53); however, issues of cost and accessibility may limit the use of such methods for the average physician.

Frank Sorvillo is an associate professor in-residence in the Department of Epidemiology at UCLA=s School of Public Health. His research interests include the epidemiology and control of infectious diseases, particularly parasitic agents.

### References

1. Cates W Jr. Estimates of the incidence and prevalence of sexually transmitted diseases in the United States. American Social Health Association Panel. Sex Transm Dis 1999;26(4 Suppl):S2-7.
2. Wolner-Hanssen P, Krieger J, Stevens CE, Kiviat NB, Koutsky L, Critchlow C, et al. Clinical manifestations of vaginal trichomoniasis. JAMA 1989;264:571-6.
3. Cotch MF, Pastorek JG II, Nugent RP, Hillier SL, Gibbs RS, Martin DH, et al. *Trichomonas vaginalis* associated with low birth weight and preterm delivery. The Vaginal Infections and Prematurity Study Group. Sex Trans Dis 1997;24:353-60.
4. Laga M, Manoka A, Kivuvu M, Malele B, Tuliza M, Nzila N, et al. Non-ulcerative sexually transmitted diseases as risk factors for HIV-1 transmission in women: results from a cohort study. AIDS 1993;7:95-102.
5. Sardana S, Sodhani P, Agarwal SS, Sehgal A, Roy M, Singh V, et al. Epidemiologic analysis of *Trichomonas vaginalis* infection in inflammatory smears. Acta Cytol 1994;38:693-7.
6. Kiviat NB, Paavonen JA, Brockway J, Critchlow C, Brunham RC, Stevens CE, et al. Cytologic manifestations of cervical and vaginal infections. 1. Epithelial and inflammatory cellular changes. JAMA 1985;253:989-96.
7. Levine WC, Pope V, Bhoomkar A, Tambe P, Lewis JS, Zaidi AA, et al. Increase in endocervical CD4 lymphocytes among women with nonulcerative sexually transmitted diseases. J Infect Dis 1998;177:167-74.
8. Fouts AC, Kraus SJ. *Trichomonas vaginalis*: reevaluation of its clinical presentation and laboratory diagnosis. J Infect Dis 1980;141:137-43.
9. Kreiss J, Willerford DM, Hensel M, Emonhy W, Plummer F, Ndinya-Achola J, et al. Association between cervical inflammation and cervical shedding of human immunodeficiency virus DNA. J Infect Dis 1994;170:1597-601.
10. Hobbs MM, Kzembe P, Reed AW, Miller WC, Nkata E, Zimba D, et al. *Trichomonas vaginalis* as a cause of urethritis in Malawian men. Sex Transm Dis 1999;26:381-7.
11. Draper D, Donohoe W, Mortimer L, Heine RP. Cysteine proteases of *Trichomonas vaginalis* degrade secretory leukocyte protease inhibitor. J Infect Dis 1998;178:815-9.
12. Wilkinson D, Abdool Karim SS, Harrison A, Lurie M, Colvin M, Connolly C, et al. Unrecognized sexually transmitted infections in rural South African women: a hidden epidemic. Bull World Health Organ 1999;77:22-8.
13. Ghys PD, Diallo MO, Ettiegne-Traore V, Yeboue KM, Gnaore E, Lorougnon F, et al. Genital ulcers associated with human immunodeficiency virus-related immunosuppression in female sex workers in Abidjan, Ivory Coast. J Infect Dis 1995;172:1371-4.
14. ter Muelen J, Mgaya HN, Chang-Claude J, Luande J, Mtiro H, Mhina M, et al. Risk factors for HIV infection in gynaecological inpatients in Dar Es Salaam, Tanzania, 1988-1990. East Afr Med J 1992;69:688-92.
15. Wiese W, Patel SR, Patel SC, Ohl CA, Estrada CA. A meta-analysis of the Papanicolaou smear and wet mount for the diagnosis of vaginal trichomoniasis. Am J Med 2000;108:301-8.
16. Madico G, Quinn TC, Rompalo A, McKee KT Jr, Gaydos CA. Diagnosis of *Trichomonas vaginalis* infection by PCR using vaginal swab samples. J Clin Microbiol 1998;36:3205-10.
17. van Der Schee C, van Belkum A, Zwijgers L, van Der Brugge E, O'Neil EL, Luijendijk A, et al. Improved diagnosis of *Trichomonas vaginalis* infection by PCR using vaginal swabs and urine specimens compared to diagnosis by wet mount microscopy, culture, and fluorescent staining. J Clin Microbiol 1999;37:4127-30.
18. Shuter J, Bell D, Graham D, Holbrook KA, Bellin EY. Rates of and risk factors for trichomoniasis among pregnant inmates in New York City. Sex Transm Dis 1998;25:303-7.
19. Bersoff-Matcha SJ, Horgan MM, Farser VJ, Mundy LM, Stoner BP. Sexually transmitted disease acquisition among women infected with human immunodeficiency virus type 1. J Infect Dis 1998;178:1174-7.
20. Cu-Uvin S, Hogan JW, Warren D, Klein RS, Peipert J, Schuman P, et al. Prevalence of lower genital tract infections among human immunodeficiency virus (HIV)Bseropositive and high-risk HIV-seronegative women. Clin Infect Dis 1999;29:1145-50.
21. Bunnell RE, Dahlberg L, Rolfs R, Ransom R, Gershman K, Farshy C, et al. High prevalence and incidence of sexually transmitted diseases in urban adolescent females despite moderate risk behaviors. J Infect Dis 1999;180:1624-31.
22. DeHovitz JA, Kelly P, Feldman J, Sierra MF, Clarke L, Bromberg J, et al. Sexually transmitted diseases, sexual behavior, and cocaine use in inner-city women. Am J Epidemiol 1994;140:1125-34.
23. Fleisher JM, Senie RT, Minkoff H, Jaccard J. Condom use relative to knowledge of sexually transmitted disease prevention, method of birth control, and past or present infection. J Community Health 1994;19:395-407.
24. Pabst KM, Reichart CA, Knud-Hansen CR, Wasserheit JN, Quinn TC, Shah K, et al. Disease prevalence among women attending a sexually transmitted disease clinic varies with reason for visit. Sex Transm Dis 1992;19:88-91.
25. Wilson TE, Minkoff H, McCalla S, Petterkin C, Jaccard J. The relationship between pregnancy and sexual risk taking. Am J Obstet Gynecol 1996;174:1033-6.
26. Miller JM, Chambers DC, Miller JM. Infection with *Trichomonas vaginalis* in a black population. J Natl Med Assoc 1989;81:701-2.
27. Cotch MF, Pastorek JG II, Nugent RP, Yerg DE, Martin DH, Eschenbach DA. Demographic and behavioral predictors of *Trichomonas vaginalis* infection among pregnant women. Obstet Gynecol 1991;78:1087-92.
28. Barbone F, Austin H, Louv WC, Alexander WJ. A follow-up study of methods of contraception, sexual activity, and rates of trichomoniasis, candidiasis, and bacterial vaginosis. Am J Obstet Gynecol 1990;163:510-14.
29. Shafer MA, Sweet RL, Ohm-Smith MJ, Shalwitz J, Beck A, Schacter J. Microbiology of lower genital tract in postmenarchal adolescent girls: Differences by sexual activity, contraception, and presence of nonspecific vaginitis. Pediatrics 1985;107;974-81.
30. Hardy PH, Hardy JB, Nell EE, Graham DA, Spence MR, Rosenbaum RC. Prevalence of six sexually transmitted disease agents among pregnant inner-city adolescents and pregnancy outcome. Lancet 1984;2:333-7.
31. Bell TA, Farrow JA, Stamm WE, Critchlow CW, Holmes KK. Sexually transmitted diseases in females in a juvenile detention center. Sex Transm Dis 1985;12:140-4.

32. McCormack WM, Evrard JR, Laughlin CF, Rosner B, Alpert S, Crockett VA, et al. Sexually transmitted conditions among women college students. Am J Obstet Gynecol 1981;139:130-3.
33. Osborne NG, Grubin L, Pratson L. Vaginitis in sexually active women: Relationship to nine sexually transmitted organisms. Am J Obstet Gynecol 1982;142:962-7.
34. Ris HW, Dodge RW. Trichomonas and yeast vaginitis in institutionalized adolescent girls. Am J Dis Child 1973;1125:206-9.
35. Ipsen J, Feigl P. A biomathematical model for prevalence of *Trichomonas vaginalis*. Am J Epidemiol 1970;91:175-84.
36. Rosenberg MJ, Davidson AJ, Chen J-H, Judson FN, Douglas JM. Barrier contraceptives and sexually transmitted diseases in women: a comparison of female-dependent methods and condoms. Am J Public Health 1992;82:669-74.
37. Wilson TE, Minkoff H, DeHovitz J, Feldman J, Landesman S. The relationship of cocaine use and human immunodeficiency virus serostatus to incident sexually transmitted diseases among women. Sex Transm Dis 1998;25:70-5.
38. Sorvillo FJ, Kovacs A, Kerndt P, Stek A, Muderspach L, Sanchez-Keeland L. Risk factors for trichomoniasis among women with HIV infection at a public clinic in Los Angeles County; Implications for HIV prevention. Am J Trop Med Hyg 1998;58:495-500.
39. Niccolai LM, Kopicko JJ, Kassie A, Petros H, Clark RA, Kissinger P. Incidence and predictors of reinfection with *Trichomonas vaginalis* in HIV-infected women. Sex Transm Dis 2000;27:284-8.
40. Krieger JN, Verdon M, Siegel N, Critchlow C, Holmes KK. Risk assessment and laboratory diagnosis of trichomoniasis in men. J Infect Dis 1992;166:1362-6.
41. Borchardt KA, Al-Haraci S, Maida N. Prevalence of *Trichomonas vaginalis* in a male sexually transmitted disease clinic population by interview, wet mount microscopy, and the InPouch TV test. Genitourin Med 1995;71:405-6.
42. Joyner JL, Douglas JM Jr, Ragsdale S, Foster M, Judson FN. Comparative prevalence of infection with *Trichomonas vaginalis* among men attending a sexually transmitted disease clinic. Sex Transm Dis 2000;27:236-40.
43. Saxena SB, Jenkens RR. Prevalence of *Trichomonas vaginalis* in men at high risk for sexually transmitted diseases. Sex Transm Dis 1991;18:138-42.
44. Grady WR, Tanfer K. Condom breakage and slippage among men in the United States. Fam Plann Perspect 1994;26:107-12.
45. Stewart DL, DeForge BR, Hartmann P, Kaminski M, Pecukonia E. Attitudes toward condom use and AIDS among patients from an urban family planning practice center. J Natl Med Assoc 1991;83:772-6.
46. Aral SO, Mosher WD, Cates W. Vaginal douching among women of reproductive age in the United States: 1988. Am J Public Health 1992;82:210-14.
47. Scholes D, Stergachis A, Ichikawa LE, Heidrich FE, Holmes KK, Stamm WE. Vaginal douching as a risk factor for cervical *Chlamydia trachomatis* infection. Obstet Gynecol 1998;91:993-7.
48. Bennett C. The black population in the United States: March 1992. Current Population Reports. Washington: US Bureau of Census; 1993. Pub. no. P20-471. p. 5.
49. Aral SO, Holmes KK. Epidemiology of sexual behaviour and sexually transmitted disease. In: Sexually transmitted diseases. 2nd edition. New York: McGraw-Hill Inc; 1989.
50. Grosskurth H, Mosha F, Todd J. Impact of improved treatment of sexually transmitted diseases on HIV infection in rural Tanzania: randomised controlled trial. Lancet 1995;346:530-6.
51. Wasserheit JN. Epidemiological synergy, interrelationships between human immunodeficiency virus infection and other sexually transmitted diseases. Sex Transm Dis 1992;19:6177.
52. Schwebke JR, Morgan SC, Pinson GB. Validity of self-obtained vaginal specimens for diagnosis of trichomoniasis. J Clin Microbiol 1997;35:1618-19.
53. Mayta H, Gilman RH, Calderon MM, Gottlieb A, Soto G, Tuero I, et al. 18S ribosomal DNA-based PCR for diagnosis of *Trichomonas vaginalis*. J Clin Microbiol 2000;38:2683-7.
